# Clinical validation of a novel bioluminescence imaging technology for aiding the assessment of carious lesion activity status

**DOI:** 10.1002/cre2.400

**Published:** 2021-03-10

**Authors:** Nigel Pitts, Neil Shanks, Christopher Longbottom, Marjory Willins, Bruce Vernon

**Affiliations:** ^1^ Dental Innovation and Impact, Faculty of Dentistry, Oral & Craniofacial Sciences King's College London Dental Institute London UK; ^2^ Downie, Harper & Shanks Dental Practice Edinburgh UK; ^3^ CALCIVIS Ltd. Edinburgh UK

**Keywords:** activity, bioluminescence, caries, lesions

## Abstract

**Objectives:**

Clinical validation of a bioluminescence imaging system (Cis) as measured by the level of agreement between clinician visual and tactile assessment of carious lesion presence and activity and the presence/absence of elevated luminescence on a tooth surface determined from intraoral image mapping.

**Materials and Methods:**

This was a regulatory clinical study designed in consultation with the FDA. The design was a prospective, five‐investigator, nonrandomized, post‐approval, clinical study utilizing the Cis to provide images of elevated calcium ion concentration (indicative of active demineralization) on tooth surfaces via use of a photoprotein. Imaged teeth were identified as “sound” or having “active lesions.” Images were scored independently for luminescence.

**Results:**

A total of 110 participants aged 7–74 years were imaged. Of the 90 teeth assessed as “sound,” 88 were deemed to show no luminescence by the reviewing investigator, a negative percentage agreement of 97.8% (significantly >50% agreement [*p* < .0001]; one‐sided 97.5% confidence interval [CI]: 0.9220). Of the 86 teeth initially assessed as having an “active lesion,” 78 were deemed to show luminescence by the reviewing investigator, a positive percentage agreement of 90.7% (significantly >50% agreement [*p* < .0001]; 97.5% CI: 0.8249). There were no patient‐related adverse events.

**Conclusions:**

Results show, with a high level of agreement, that Cis can differentiate tooth surfaces clinically identified as involving active enamel lesions (ICDAS code 2/3), from sound sites (biochemically equivalent to inactive lesions) and that the system is safe for clinical use.

## 1 INTRODUCTION

Dental caries (tooth decay) prevalence varies worldwide from 15 to 84% and while currently declining, it is still a significant clinical and public health problem (Frenken et al., 2017; Pitts et al., 2017). The development of caries lesions involves a net mineral loss from dental hard tissue, mediated by acid diffusing from bacterial dental plaque on tooth surfaces. This localized demineralization may, or may not, lead to progressive loss of tooth structure and associated pain and morbidity (Pitts et al., 2017; Selwitz et al., 2007).

Detecting, assessing, diagnosing, and treating carious lesions is a core activity in dentistry (Fejerskov et al., 2008; Paris et al., 2013; Pitts, 2009). The main detection and diagnostic aids have long been visual assessment of subtle differences in optical and physical characteristics of the lesion surface, including the use of a blunt ball‐ended probe, together with radiographs (Neuhaus & Lussi, 2018). Assessment of a lesion's “true” activity status in a single visit remains challenging and determination of which “white spot” lesions will progress toward cavitation can currently only be validated prospectively by serially monitoring, over a specific time period, which lesions eventually undergo cavitation (Ekstrand & Martignon, 2013; Neuhaus & Lussi, 2018; Nyvad et al., 1999).

Several technologies can aid caries lesion detection; however, determination of the activity status of an individual lesion (one with ongoing net demineralization) is also required to fully assess treatment needs and success. Current methods of activity assessment are problematic and involve the dental professional's subjective judgment (Neuhaus & Lussi, 2018). There is therefore a need to develop a technique to aid in identifying the activity status of caries lesions so as to optimize the use of noninvasive preventive therapies, as compared to operative (surgical removal of dental tissue) interventions that tend to be more expensive and can have long‐term negative clinical consequences, such as entry into the “restoration–rerestoration cycle” (Meyer‐Lueckel et al., 2013; Schwendicke et al., 2018) as well as requiring the production of aerosols in a post COVID‐19 pandemic world.

Whilst a number of different technologies, such a quantitative light fluorescence (QLF) and polarization‐sensitive optical coherence tomography (PS‐OCT), have shown indications that they may have some limited value in monitoring lesion activity, these have mostly been in a laboratory setting, that is, in vitro, and none is yet in general use in vivo (Fried, 2019; Kim, 2019).

A novel bioluminescence technology, the CALCIVIS Imaging System, has been developed to aid in the assessment of caries lesion activity. An in vitro study on occlusal sites of extracted teeth, using a pH dye for validation of activity, demonstrated: (a) a sensitivity of 92.5%; (b) a specificity of 90%, and (c) a diagnostic accuracy for activity assessment of caries lesions which was not significantly different from the value obtained using the ICDAS activity assessment method (Jablonski‐Momeni et al., 2018), which was the validated activity assessment method used in the current article—vide infra. The technology underlying the CALCIVIS imaging system was developed to help address this unmet need. It combines a sensitive intraoral imaging device and a photoprotein solution that produces luminescence, or light emission (low level in the visible spectrum), in the presence of calcium ions in solution, as they are released from actively demineralizing regions of a tooth surface, that is, in the enlarged openings of the enamel pores of the surface zone of an enamel lesion. The biochemical reaction is provided below (Longbottom & Vernon, 2019).

$$3\times {\mathrm{Ca}}^{2+}+\text{photoprotein}=\mathrm{Apo}-\text{protein}+\text{Cofactor}+{\mathrm{CO}}_{2}+\text{blue light}\;\left(\text{in region of}\;480\;\mathrm{nm}\right).$$

The light emission results from a chemical reaction between the photoprotein and free calcium ions, in contrast to fluorescence‐based technologies, which require an excitatory light source (Neuhaus & Lussi, 2018). The majority of the emitted light signal is captured and recorded by an intraoral camera in less than 0.1 s. System‐embedded software precisely overlays black and white and luminescence images of the tooth surface, highlighting locations where elevated levels of calcium ions are present and providing “active demineralization maps” of the tooth surfaces, which can then be interpreted by the dental professional (CALCIVIS, 2019; Longbottom & Vernon, 2019). By definition, the caries process involves the loss of mineral ions, chiefly calcium and phosphate ions, from the enamel structure and the presence of elevated levels of calcium ions in the surface enamel pores *ipso facto* is an indicator of caries lesion activity. ex vivo research on freshly extracted and stored teeth has demonstrated a strong correlation between positive light signals generated by the CALCIVIS imaging system and caries lesion activity status (as assessed by visual and tactile criteria) (Jablonski‐Momeni & Kneib, 2016a, 2016b). This has the potential to aid in determining management options for each caries lesion.

### 1.1 Context for studies of carious lesion activity

One time‐point in vivo assessment of the activity status of a lesion using a visual‐tactile method is generally recognized as being subjective and is limited by the examiner's visual acuity and tactile sensitivity. One of the visual‐tactile methods with supporting peer‐reviewed publications, the Nyvad criteria (Nyvad & Baelum, 2018; Nyvad et al., 1999, 2003), for example, when used by highly trained and calibrated examiners, was shown to have a reliability of 68.7% when used to designate noncavitated lesions as “active” and 72.5% to designate noncavitated inactive lesions—meaning that there was between a 1‐in‐3 and 1‐in‐4 chance that a lesion “active” or “inactive” designation would “transition” to the other designation when the same lesion was examined subsequently by the same examiner. Additionally, analysis of the results of a large scale 3‐year longitudinal study, which have been used to “validate” the Nyvad criteria, shows that in the control group (observation‐only data) for this method of designating inactive and active lesions (from baseline data to 3‐year data) has values for sensitivity, specificity, positive predictive value and negative predictive value of 65; 46; 53 and 59%, respectively, for occlusal sites (when the 3‐year outcome threshold was placed at the cavitation/filling level). Thus, all four of these validity parameters have values that are well below the generally acceptable level for a medical diagnostic device.

Therefore, attempting to measure the performance of any technology designed to aid this diagnostic task of lesion activity assessment by using one of the current visual‐tactile methods as a reference point (or “Gold standard”) presents a substantial methodological challenge. As noted above, the current visual/tactile methods of lesion activity assessment, such as the Nyvad method (or the ICDAS activity assessment technique (Ekstrand et al., 2009)), fall considerably short of acceptable accuracy, hence, any study which uses either of these methods as a *simple* “gold standard” one time‐point validation method faces producing unknowable levels of false positive (FP) and false negative (FN) results for any new technique under investigation. Thus, using such a simple comparative methodology, any new technology can only ever be as good as or worse than, in terms of accuracy, the supposed “gold standard.” Hence, such a methodology is an exercise in futility—why carry out such a study when the test technique cannot possibly be shown to improve upon the knowingly “invalid”/“limited” reference standard?

The crux of the problem of determining as accurately as possible the “true” gold standard “activity status” of a lesion in vivo lies in a methodological paradox. If a new test technology operates at a molecular/ionic level, independent of the macro‐morphology of the enamel, as is the case with the Calcivis technology, using as a reference/validation standard a method which operates at a macro‐morphological level (i.e., visual‐tactile) means that the new technology is operating at resolution which is several orders of magnitude greater sensitivity than the reference method—the enamel pores of an active lesion are of the order of hundreds of nanometers to a micron in diameter (Haikel et al., 1983; Thylstrup et al., 1983) well beyond the limit of the human visual acuity used in the visual‐tactile method.

All this demonstrates that there is a major methodological challenge in attempting to validly test the performance of the Calcivis imaging system in aiding the identification of active lesions compared with nonactive surfaces.

However, for an initial assessment, it is possible to reduce the probability of these unknown “validator errors” risks occurring by using, as comparative test samples, tooth surfaces which are known, from large numbers of epidemiological studies, to have the maximum difference in probability of exhibiting active caries lesions. Such sites in permanent teeth are: for least probability sites, the buccal surfaces of canines (or incisors) and for highest probability sites, the occlusal (pit and fissure) surfaces of molars (and premolars) (Batchelor & Sheiham, 2004; Massler et al., 1954; Poulsen & Horowitz, 1974). Thus, designation by a clinician (using visual/tactile criteria) of a site on the buccal surface of a canine (or incisor) as “sound” has the highest probability of being correct (since it has the lowest probability of exhibiting an active lesion); similarly, the designation of a lesion on a pit and fissure site on a molar (or premolar) as an “active lesion” has the highest probability of being correct. Hence, by using these two sample types as the validation sites, the probability of producing both FP and FN results is minimized, albeit to a currently unknowable extent. It is, of course, possible that the buccal surface of a canine (or incisor) might be undergoing demineralization at a subclinical level but that probability is small. Equally, it is possible that a lesion designated as active by a clinician, using subjective assessment methods, may in reality have become inactive, but, by using the pit and fissure sites of molars (and premolars), that probability is minimized.

It should be noted at this point that, in terms of the degree (or level) of release of calcium ions from the enamel surface, a sound site is *by definition identical* to an arrested/inactive lesion site, since neither is undergoing net mineral loss, even though the macroscopic clinical appearance will be considerably different. This equivalence is key to the understanding of the methodological approach taken in this study.

To reiterate, in an ideal world, testing the performance of a new technology for its ability to determine if a lesion is active or arrested (inactive) would involve comparing lesions which are known to be definitely active with those which are known to be definitely arrested. However, because there is as yet no identified clinical or technological (in vivo) gold standard validation method for determining lesion activity to a high degree of accuracy, this ideal comparative methodology is not possible in any meaningful sense, due to the considerable limitations of the current clinical validation techniques. The use of such a clinically designated “active” versus “inactive” lesions comparison methodology is thus totally inappropriate and would beg the question “Is the new technology more accurate than the validator?”—analysis of the results could not determine which of the techniques—test or validator—is closer to identifying the “true” activity status of each lesion.

Thus, for the current study to validate this novel bioluminescence technique based on the identification of the presence of calcium ions in the surface enamel, the authors chose to use the sampling method described above, that is, using specific tooth sites where: (a) there is a high probability that a clinically designated “active” lesion is indeed active (an identified white spot lesion in a plaque stagnation area) and (b) there is a high probability that a clinically designated sound site is indeed sound (which is equivalent, in terms of the surface enamel pore fluid concentration of calcium ions, to an arrested lesion). By this means, together with specific training of the investigators (in the identification of active lesion and sound sites) and the independent review of the study images, the authors sought to minimize the probability of validator error.

The use of a comparison of two different tooth sites raises a potential issue with examiner bias in assessing the images, since the independent (second) examiners could infer that the smooth surfaces had been classified as sound and occlusal surfaces had been assessed as exhibiting an “active lesion” by the first examiner. This potential bias issue is dealt with in Section 4.

This study was required for submission to the United States regulators and therefore the study design was agreed in advance with the US FDA prior to local approvals. It followed a smaller pilot clinical study, and the completion of toxicological and biocompatibility testing as required by the regulators.

The purpose of this current prospective, multisite, nonrandomized, post‐approval, clinical study was to assess the level of agreement in carious lesion activity status between the findings of general dental practitioners' visual and tactile assessment with those of the Calcivis imaging device in two teeth populations (i.e., with and without visible lesions).The performance of the device was measured by presence or absence of elevated luminescence, determined from intraoral image mapping of teeth surfaces with or without a visible lesion. Any adverse events were also collected.

## 2 MATERIALS AND METHODS

This was a prospective, five‐investigator, nonrandomized, post‐approval, clinical study conducted to the highest of standards for medical device studies. All five Investigators were fully qualified (UK General Dental Council registered) and experienced Dental Surgeons (minimum BDS), working as principles/partners in four general dental practices in Scotland. The Chief Investigator was Neil Shanks, one of the manuscript authors. The final study plan was approved by South East Scotland Research Ethics Committee 02 (REC Ref: 15‐SS‐0231; approval granted January 28, 2016) in accordance with ethical principles outlined in the Declaration of Helsinki; the European Standard of BS EN ISO 14155:2011: Clinical investigation of medical devices for human subjects—good clinical practice; the International Conference on Harmonization Good Clinical Practice Guidelines; and STROBE guidelines. In addition, local NHS Health Board approval was also obtained. There were four nonsubstantial amendments to the clinical study plan, all of which occurred prior to study start, did not affect study flow or outcomes, and received ethical committee approval.

### 2.1 Participants

Eligible participants ≥6 years old had at least one unrestored, accessible, free, smooth buccal surface on a canine or incisor, away from the gingival surface, with no visible lesion (coded ICDAS 0) and/or one unrestored, accessible, erupting or erupted molar or premolar with a visible initial lesion (coded ICDAS 2 or 3) on the occlusal surface, in a plaque stagnation area. The only exclusion criteria were: pregnancy; breastfeeding; tooth bleaching within 2 weeks of imaging; ongoing remineralization treatment; fixed orthodontic appliances; and currently taking part, or within 3 months of taking part, in a clinical study. The “within 2 weeks of bleaching” limitation was applied in order to limit any possible interference with the calcium ion dynamic equilibrium in the enamel pores resulting from any bleaching procedure, which might affect the Calcivis system.

### 2.2 The CALCIVIS imaging system

**FIGURE 1:**
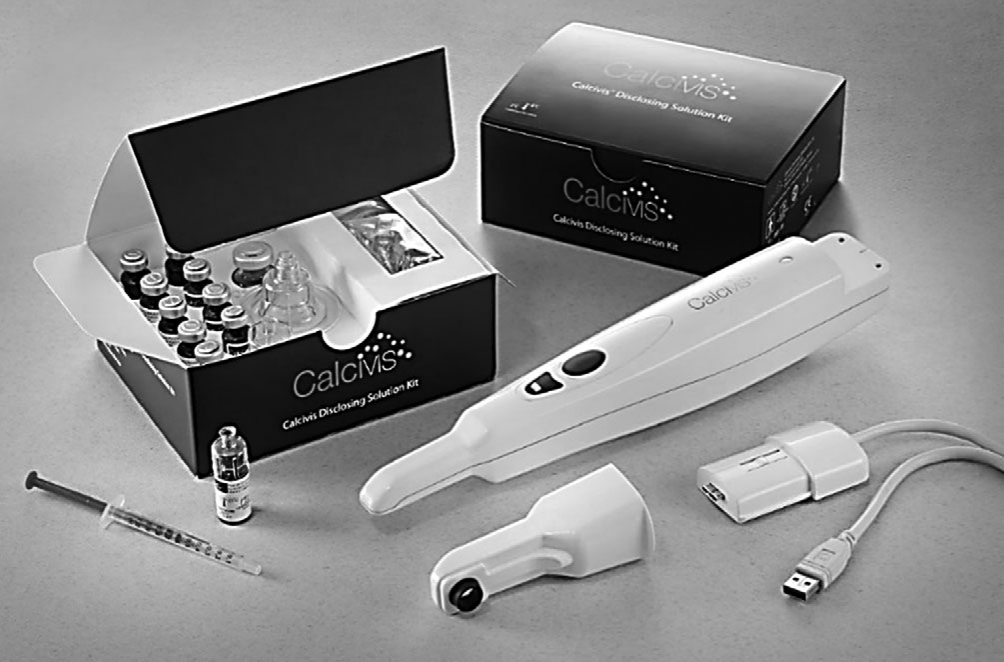
The pre‐commercial CALCIVIS imaging system (as used in the study)

The CALCIVIS imaging system consisted of an intraoral device and cradle; single‐use sterile device syringes; vial adaptors; and single‐use applicators (Figure 1; Perfect et al., 2012; Perfect & Longbottom, 2008). The CALCIVIS photoprotein was supplied freeze dried in a vial; once reconstituted, the multiuse vial was stored at 2–8°C, for a maximum of 4 weeks. Investigators were supplied with a laptop with dedicated software and instructions for use. Investigators and dental nurses were fully trained, prior to study start, on device use, with training in CALCIVIS image interpretation for the investigators only. Training was carried out by BV. Training on ICDAS staging and activity status was carried out by an expert cariologist (CL). This training involved both theoretical and practical training on extracted teeth displaying lesions across the different ICDAS staging range. Documented training took place over a number of months, with Investigators and Nurses signed off by the trainers, once an acceptable level of competence was achieved. In relation to the identification of sound sites and active noncavitated lesions it is important to note that since the use of the *full range* of the ICDAS codes was not part of this particular study the conventional assessment of intra and interexaminer reproducibility of the full ICDAS codes range was inappropriate and unnecessary—the key element required was calibration with the trainer in the identification of sound sites, that is, ICDAS code 0 surfaces, and active lesion sites which were noncavitated (at the macroscopic level), that is, ICDAS codes 2–3 lesions. Training in the identification of lesions with all the various ICDAS codes was carried out before the trainer, *individually* with each investigator, used a randomly selected 10 teeth from a pool of more than 40 teeth which displayed sound sites and noncavitated lesion sites and asked each investigator to code all of the five (posteriors) or four (anteriors) surfaces on each tooth and to assess whether any lesions were active or arrested, with a minimum of 80% agreement (for total number of the surfaces assessed) with the trainer on lesion presence/activity or sound status being required to achieve competence.

Because of the generally recognized issue of relatively lower intraexaminer reproducibility regarding ICDAS code 1 lesions compared to those for code 2 and 3 lesions in this study, in order to minimize/eliminate that potential problem, the examiners were asked to identify ICDAS code 2 or 3 lesions when seeking active lesions to image.

### 2.3 Study visits and clinical procedures

**FIGURE 2:**
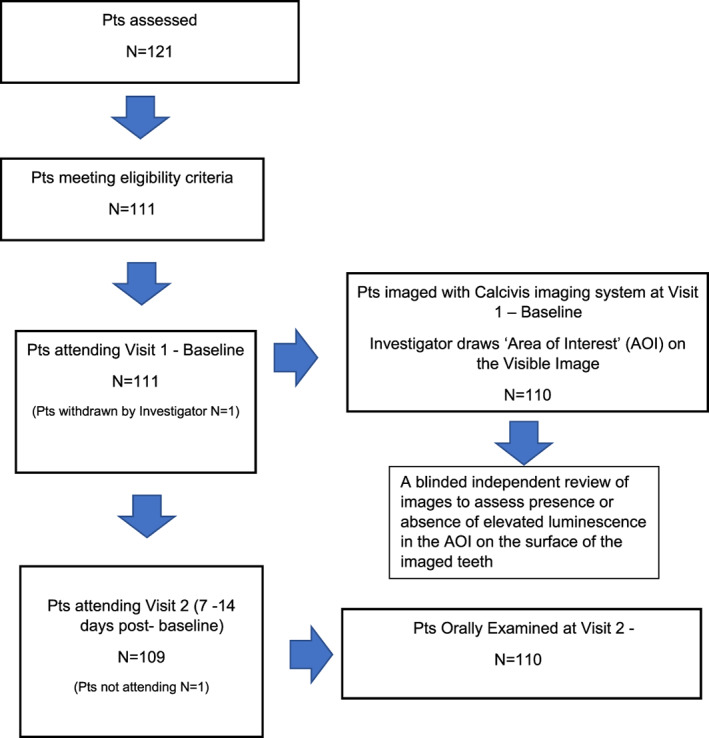
The outline of the study design, showing the flow of the patients' (Pts) experiences

The outline of the study design is shown in the Flow chart in Figure 2. Participants were identified by the investigators during routine dentist visits as meeting the inclusion/exclusion criteria and provided with study details. If they agreed to participate, they returned to the dental practice for Visit 1, where they provided written informed consent prior to entering the study. For participants aged 6–15 years, written consent was obtained from the parent or guardian. Details of participant's demographics, relevant medical history, medications, and oral hygiene regimen were recorded. The first participant took part in the study on January 14, 2017; the last participant completed the study, including safety follow‐up, on May 11, 2017.

The CALCIVIS imaging system was prepared according to manufacturer's instructions. Teeth were identified and recorded for assessment as per inclusion/exclusion criteria. A maximum of one tooth from each category (sound tooth; tooth with active noncavitated lesion) per participant was imaged. If a participant had more than one tooth in a category, the investigator used clinical judgment to choose the tooth that most clearly fitted the criteria.

All tooth surfaces to be imaged were cleaned by the investigator by brushing with nonfluoridated water or dental paste, then rinsing with an air–water spray from a conventional dental 3‐in‐1 device. No specific dental paste was recommended, if used, the choice was at the discretion of each investigator. Participants then rinsed thoroughly with nonfluoridated tap water. Following thorough air‐drying of each tooth surface to be imaged, the investigator took a color photograph with a standard intraoral camera (CareStream CS1500—factory settings) for reference and recorded the ICDAS score and lesion activity status.

**FIGURE 3:**
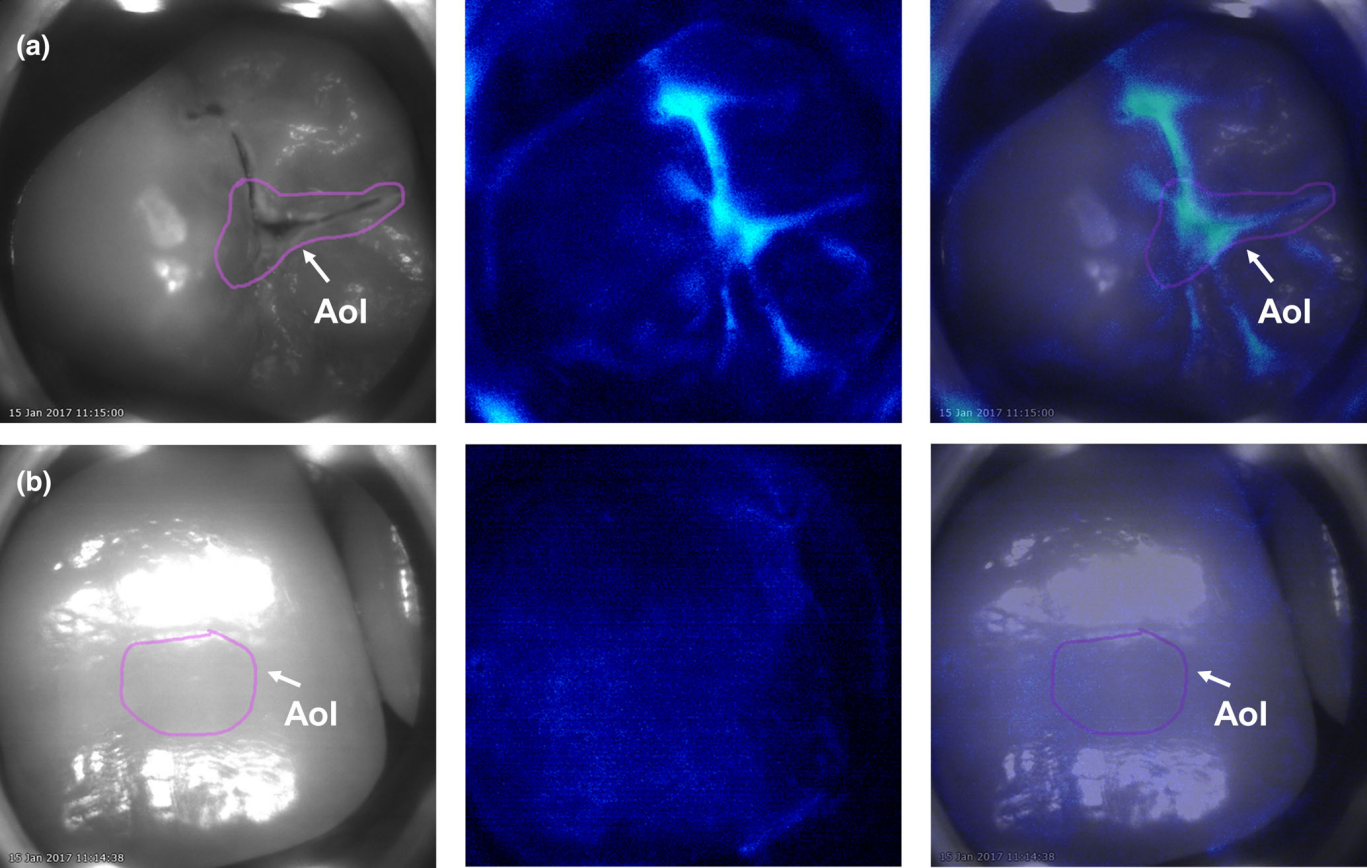
Examples of black and white, luminescence and combined images of teeth with (a) an active lesion (true positive), and (b) no lesion (true negative). The pink outline in columns 1 and 3 are the area of interest (AOI) delineated by the investigator

In preparation for use of the CALCIVIS imaging system, care was taken to ensure the tooth surface and surrounding area were free from saliva using appropriate moisture control aids (buccal dry‐guards, dry tips and saliva‐ejectors). Each tooth was then air‐dried for 5–10 s. The investigator instigated the image‐capture procedure. An image of the chosen tooth was first captured as a visible, black and white, light image, then the photoprotein was applied, followed by capturing the luminescence signal (Figure 3). The system is fully automated so that both images are taken within less than 0.5 s, including automated photoprotein application; most of the emitted light signal is captured in less than 0.1 s. The participant rinsed out with nonfluoridated tap water after imaging was completed.

Images were stored digitally on a provided laptop. The investigator was unable to see the luminescence images until they had drawn an “area of interest” on the visible image (Figure 3), based on their clinical visual/tactile examination to determine the tooth classification. The software overlaid the two sets of images (black and white and luminescence) resulting in an “active demineralization map” of each imaged tooth (Figure 3). The investigator shared the images with the participant. The investigator could provide caries preventive advice to the participant and/or parent or guardian if needed; however, the investigator would not suggest further dental treatment based on image results alone.

Visit 2 took place 7–14 days after Visit 1, when an oral examination was performed by the investigator.

### 2.4 Independent investigator review

After all images were taken and verified as acceptable by each first (originating) investigator, an independent review of the images was undertaken by one of the other study investigators to assess presence or absence of elevated luminescence on the surface of the imaged teeth. The determination by each independent reviewer was made off‐site with no input from the originating investigator. The reviewing investigator was provided with the laptop with the CALCIVIS imaging system images (visible, luminescence, and overlay; Figure 3) and the reference intraoral color images, along with the following information for each tooth: tooth ID and surface; image number; photographic evidence of “area of interest” (lesion or the sound area: Figure 3, column 1). The independent reviewers were blinded to the first (originating) investigator's recorded ICDAS scores/activity status.

The designation of which independent reviewer assessed the images from each first investigator is shown in Table 1.

**TABLE 1:** Designation of which independent reviewer assessed the images from each original investigator

Site	Original investigator	Independent investigator
1	NS (site 1)	SM (site 4)
1	ED (site 1)	AN (site 5)
3	FM (site 3)	ED (site 1)
4	SM (site 4)	NS (site 1)
5	AN (site 5)	FM (site 3)

### 2.5 Safety

Any adverse events were recorded at both visits (v1—baseline imaging visit and V2–7 to 14 days post‐imaging). If appropriate, a color image was taken of the adverse event.

### 2.6 Statistical analysis

The study sample size was calculated in terms of number of teeth of each tooth population required. As no more than one tooth of each population was imaged in each participant, the number of participants required was at most the total number of teeth required. To ensure poolability of data from each investigator, up to 36 participants were recruited to the study by each of the five investigators to obtain a minimum of 17 evaluable images of each tooth population (“sound,” “active lesion”) per investigator, after which recruitment was stopped.

For sample size calculations, the percentage agreement for each of the two tooth populations were jointly considered as measures of agreement. That is, the study was deemed a success if percentage agreement in both tooth status populations was statistically significant at the 2.5% level when compared to chance agreement (50%). To achieve at least 90% power overall, a power of 94.9% was used for each tooth status population individually. The planned method of analysis was an exact binomial test that was used to derive a required sample size of 81 for each of the tooth status populations, this being the first sample size after which all subsequent sample sizes provided at least 94.9% power. The null and alternative hypotheses were: H_0_: *p*_a,i_ = .5 versus H_1_: *p*_a,i_ > .5 for each tooth status population (where *p*_a,i_ is the percentage agreement in tooth status population). All statistical analysis was performed using SAS version 9.2 (SAS Institute, Inc., Marlow, UK).

CALCIVIS imaging system performance was measured by presence/absence of tooth surface luminescence, as assessed via expected level of agreement between the CALCIVIS imaging system and investigator rating of suitable teeth in two teeth populations: “sound teeth,” and “teeth with active noncavitated lesions,” based on previous study data and expert opinion (data on file). “Sound teeth” were expected to correspond to “no luminescence” according to the CALCIVIS imaging system in at least 70% of cases; “teeth with active lesions” were expected to correspond to “luminescence” in at least 70% of cases. CALCIVIS imaging system safety was measured by the collection of adverse events.

All statistical tests were conducted one‐sided with a 2.5% level of significance; no adjustment was made for multiple testing. The percentage agreement of the first investigator's ICDAS activity score and the reviewing investigator's CALCIVIS finding (“no luminescence” or “luminescence”) was presented along with an exact one‐sided 97.5% confidence interval (97.5% CI) and *p*‐value comparing the percentage agreement to 0.5 for teeth rated with “no visible lesion” that were assessed as “no luminescence” by the CALCIVIS imaging system or teeth rated with an “active lesion” that were assessed as displaying “luminescence” by the CALCIVIS imaging system.

The analysis of agreement was performed on the Agreement Population, which included all teeth on which there was an ICDAS score by the first investigator. Luminescence data was collected and analyzed from the reviewing investigator only. Missing data arising from teeth where the CALCIVIS imaging system assessment was uninterpretable for reasons unrelated to the assessment outcome were not imputed. Adverse events were summarized descriptively for the safety population, which included all those on whom the CALCIVIS imaging system was used.

## 3 RESULTS

Of 121 eligible participants, 111 were recruited of which 110 were imaged with the CALCIVIS imaging system. There were 61 males (55.5%) and 49 females (44.5%) in the Safety population, with an age range of 7–74 years (mean 24.3 years; *SD* 12.22).

### 3.1 Teeth/image eligibility

**FIGURE 4:**
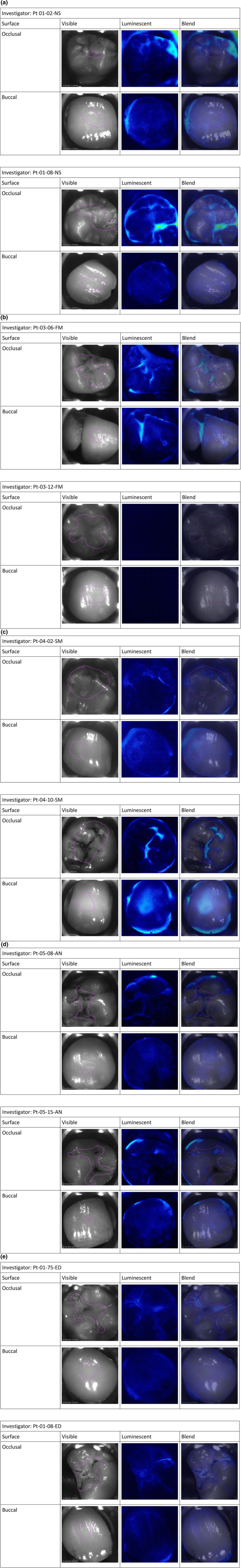
This montage displays sets of paired (occlusal and buccal) images from the two different subjects for each of the 5 examiners. From left to right, the images are Visible (V), Luminescence (L) and Blended (B) images, with each ‘area of interest’ (AOI) outlined in purple in the V and B images. Figures 4a‐4e are displayed over this page and the following 4 pages. 4a: Examiner NS: Pt 01‐02 (occlusal) the AOI was drawn over part of the fissure pattern, the luminescence and blended images show no significant luminescence in the AOI. For the buccal image the AOI was in the middle third of the surface, with generalised ‘amorphous’ background luminescence in the L and B images. For Pt 01‐08 (occlusal) the AOI had elevated luminescence visible in both of the separate aspects of the fissure pattern. For the buccal image the AOI had generalised ‘amorphous’ background luminescence. 4b: Examiner FM: Pt 03‐06 (occlusal) the AOI had elevated luminescence visible in most aspects of the fissure pattern. For the buccal image the AOI had irregular ‘amorphous’ background luminescence. (The elevated luminescence towards the left of the image is almost certainly saliva pooling in the interstitial space). For Pt 03‐12 (occlusal) the AOI had no elevated luminescence visible in any aspect of the fissure pattern. For the buccal image the AOI had no luminescence. 4c: Examiner SM: For Pt 04‐02 (occlusal) the AOI had elevated luminescence visible in the distal fissure pattern in the L and B images. For the buccal image the AOI had a minimally elevated irregular ‘amorphous’ luminescence. For Pt 04‐10 (occlusal) the AOI had elevated luminescence visible in most aspects of the fissure pattern. For the buccal image the AOI was associated with a white area of the enamel, visible in the V image, with elevated luminescence in the L and B images in the AOI. 4d: Examiner AN: For Pt 05‐08 (occlusal) the AOI had elevated luminescence visible in most aspects of the fissure pattern. For the buccal image the AOI had no luminescence. For Pt 05‐15 (occlusal) the AOI had elevated luminescence visible in three distinct aspects of the fissure pattern. For the buccal image the AOI had a minimally elevated irregular ‘amorphous’ luminescence. 4e: Examiner ED: For Pt 01‐75 (occlusal) the AOI had elevated luminescence visible in several aspects of fissure pattern. For the buccal image the AOI a minimally elevated irregular ‘amorphous’ luminescence. For Pt 01‐08 (occlusal) the AOI had elevated luminescence visible in two separate aspects of the fissure pattern. For the buccal image the AOI had a minimally elevated irregular ‘amorphous’ luminescence

Examples of black and white, luminescence and combined images are shown in Figures 3 and 4. There was a total of 96 sound teeth and 96 active lesion teeth. From these, 16 images could not be used: six were uninterpretable; eight were not saved; two were excluded due to premature or accidental device firing, resulting in 90 images of sound teeth with no visible lesions (ICDAS 0) and 86 images of teeth with an active lesion (ICDAS 2 = 51; ICDAS 3 = 35). Figure 4 comprises a montage of two sets of captured images for each of the five examiners in the study, each set illustrating “paired” occlusal and smooth surface images, each set obtained from a different subject. These sets of images each show the slight variation in the level of the “background luminescence” which generally occurs between teeth but also shows the visually obvious difference between a lack of signal from clinically designated sound surfaces compared with the strong luminescence signal from active lesions. Exceptions to this stark difference in the level of luminescence signal are image 4c Pt‐04‐10‐SM buccal, which illustrates a false negative surface “sound” clinical designation and images 4b Pt‐03‐12‐FM occlusal, which show a false positive “active lesion” clinical designation.

### 3.2 Analysis

Table 1 shows which independent reviewer assessed the images from each original investigator. Table 2 shows the results for the originating investigators' assessments of ICDAS scores and the reviewing investigators' interpretations of the Calcivis system images. All lesions with ICDAS scores 2 and 3 were assessed by the originating investigators as active lesions.

**TABLE 2:** Originating investigators' assessments of ICDAS scores (left column) and reviewing investigators' interpretations of the Calcivis system images: Primary analysis agreement population

ICDAS	As interpreted by the Calcivis system			
	No luminescence	Luminescence	Missing	Total
ICDAS 0	88	2	0	90
ICDAS 2	7	44	0	51
ICDAS 3	1	34	0	35
Total	96	80	0	176

*Note*: All results pooled. All lesions with ICDAS scores 2 and 3 were assessed by the originating investigators as active lesions.

Of the 90 sound teeth with no visible lesion, as assessed by all the first investigators using ICDAS, 88 were deemed by the reviewing investigators to show no luminescence, a negative percentage agreement for true negatives of 97.8%. This was statistically significantly greater than 50% agreement (*p* < .0001) with a 97.5% CI of 0.9220.

Of the 86 teeth assessed by all the first investigators as having an active lesion, 78 were deemed to show luminescence by the reviewing investigators, a true positive percentage agreement of 90.7%. This was statistically significantly greater than 50% agreement (*p* < .0001) with a 97.5% CI of 0.8249.

### 3.3 Safety

There were no adverse events related to either the device or photoprotein.

## 4 DISCUSSION

The accurate assessment/identification of caries lesion activity is one of the key elements in caries lesion diagnosis and management (Pitts et al., 2017). The World Dental Federation (FDI) has, in September 2019, published a Policy Statement on *Caries Lesions and First Restorative Treatment* (FDI, 2019)—this seeks to “encourage a shift from a restorative approach to caries management to the delivery of preventive dental medicine.” It specifically recommends that any tissue removal decision must consider both lesion *stage* and *activity*. The current conventional means of assessing lesion activity is to use sequential assessments of a detection system (visual or technology‐based), such as ICDAS coding or radiography, and compare the size/depth of a lesion over time. This involves inherent disadvantages, such as difficulties in standardization issues and the relatively long timeframe needed between assessments, in addition to the risk of leaving untreated an active lesion (Pitts, 2004).

The surface of a sound enamel site is essentially “smooth” with small pores at the submicron level of imaging, whereas an active caries lesion displays a “roughened” surface with enlarged enamel “pores,” termed “focal holes.” An arrested lesion, as a result of a combination of abrasion of the roughened surface and redeposition of mineral into/onto the surface porosities of the lesion, is essentially smooth but with a slightly disrupted surface. While it is relatively straightforward for a dental professional to differentiate between a sound enamel surface and one with a distinct white spot lesion, it is difficult to differentiate objectively between the optical properties of an active and an arrested white spot lesion, with only subtle and minor differences in quantity and quality of enamel surface light reflectivity (Fejerskov & Larsen, 2015). Similarly, using a 0.5 mm ball‐ended metal probe as a vibration‐assessment tool in an attempt to detect micron level changes in surface morphology are inherently highly subjective.

The CALCIVIS imaging system was developed to aid in the characterization step of assessment, when a potential carious lesion has already been detected, or suspected, and more information is needed about that lesion regarding its current activity status in relation to ongoing calcium ion loss. In the context of testing the CALCIVIS imaging system, a clinically categorized sound enamel surface is equivalent/identical to an arrested caries lesion. This is due to no net loss of calcium ions such that the average mineral density (or, the obverse, porosity) of the surface enamel layer at a sound site is effectively identical to that of an arrested lesion, even though the appearance can be quite different between translucent sound enamel and a white/opaque underlying (demineralized) enamel (carious) lesion (Fejerskov & Larsen, 2015).

The CALCIVIS imaging system is novel and without current precedent in clinical dentistry. While systems such as radiology, fluorescence‐based technologies and transillumination may help with lesion identification, no currently used technology allows direct visualization at one specific time‐point of calcium ions as an indicator of ongoing demineralization, hence the activity status of caries lesions. The results of the comparisons in this study showed that use of the CALCIVIS imaging system correctly identified 90.7% of lesions designated as “active” by the first investigator (true positives), with 9.3% of these lesions producing an “apparent” false negative result. The CALCIVIS imaging system correctly identified 97.8% of the sound sites (true negatives), with 2.2% of sites apparent false positive results. Even though the CALCIVIS imaging system is not a caries *detection* device, few, if any, lesion detection‐only techniques reach such high levels (summarized by Neuhaus & Lussi, 2018) as the above true positive and negative rates for the CALCIVIS imaging system in this study.

The apparent false negative results, where there was a lack of luminescence from sites designated as active lesions, may have been due to incorrect activity status designation by the first investigators, given the widely recognized subjective nature of the clinical‐visual assessment of enamel lesion activity status. In the “real‐world,” the dental professional will integrate CALCIVIS imaging system test results with clinical knowledge, including patient caries risk, and interpret the results according to the total clinical picture, thus the 9% false negative figure is likely to be an overestimate of the “true” percentage false negative value and, with a risk‐informed recall regime in place, will be modulated through reimaging within a risk‐appropriate timeframe. In relation to the 2% false positive rate, in the context of “real‐world” practice, the clinical impact of such a low false positive error will be minimal, since preventive measures for active enamel lesions are nondestructive, hence no irreversible operative treatment decision for an inactive lesion would be involved.

Theoretically, there would appear to be an issue of potential bias in the methodology, since, by comparing two different tooth surface types, the independent examiners could infer in advance the designated status (“sound” or “active lesion”) of each surface (occlusal or free‐smooth). However, the status designation was arrived at using a CLINICAL VISUAL assessment, whereas the luminescence images are created *independently* of the macro‐morphological characteristics of the enamel surface which are assessed when using clinical visual‐tactile methods. The images in Figure 4 illustrate the striking differences between the luminescence levels obtained from the “areas of interest” in designated sound surfaces and a designated active lesion surfaces—these optical signal differences are so large that even though the examiners might be aware of the prior designated status of each surface, the visual signals are so starkly different that this would override any potential bias—see Figure 4. It is of note in Figure 4 that the “background” levels of luminescence intensity from the nonfissure aspects of the occlusal sites are generally at the same levels as those from across the whole of the free‐smooth surface sites in the same subjects. The fact that several “false negative” and two “false positive” results for the status designations were actually obtained, despite this prior awareness, indicates that any potential bias effect was overridden by the compelling visual evidence of the extent of the luminescence (optical) signal.

Potential confounders of the Cis include two obvious sources of calcium ions: saliva and the dental biofilm (plaque)—these factors can be effectively controlled using standard conventional clinical techniques (of isolation, air‐drying and cleaning) which form part of the Cis pre‐imaging protocol.

These study results demonstrate that the CALCIVIS imaging system can potentially provide clinicians with a clinical tool that is the first to allow them to visualize active ongoing demineralization to aid the identification of the activity status of caries lesions. The use of such a device could greatly improve accurate and efficient targeting of secondary preventive measures such as pit and fissure sealants, topical fluorides, remineralizing technologies and oral hygiene measures for specific tooth sites. Information from the CALCIVIS imaging system is potentially valuable in the context of a systematic approach to caries detection and assessment such as the ICDAS (International Caries Detection and Assessment System), which underpins the American Dental Association's Caries Classification System (International Caries Detection and Assessment System Coordinating Committee, 2011; Young et al., 2015). Dental professionals will be able to integrate CALCIVIS data with clinical data on probable depth and surface morphology status of a lesion. For example, whereas ICDAS code 2 lesions have a noncavitated surface at the macroscopic level, ICDAS code 3 lesions have a microcavitated surface (Young et al., 2015). CALCIVIS data, and other data, such as the patient's caries‐risk category, could be combined with these scores to determine the optimum treatment option for lesions with such codes.

In conclusion, the results of this clinical study show that the CALCIVIS imaging system can be used to visualize active demineralization sites in clinically identified enamel lesions and is safe for clinical use.

### CLINICAL SIGNIFICANCE

The study results indicate that the Cis provides a highly accurate one time‐point aid for clinicians in determining the presence of elevated calcium ion concentrations (indicative of activity status) of clinically detected enamel caries lesions compared to healthy sites (caries inactive)—this differentiation between active and inactive sites is of critical importance in deciding the optimum treatment/therapy for individual enamel lesions.

## AUTHOR CONTRIBUTION

All authors contributed to the study design and interpretation and to drafting the manuscript, including final approval. NP, CL, MW and BV also contributed to the conception of the study; NS also contributed to data acquisition

## CONFLICT OF INTEREST

Christopher Longbottom reports personal fees from Calcivis Ltd., during the conduct of the study; personal fees from Calcivis Ltd, outside the submitted work; In addition, Christopher Longbottom has a patent No. WO2008075081A2 issued. Nigel Pitts reports personal fees from Calcivis Ltd., outside the submitted work. Neil Shanks has nothing to disclose. Marjory Willins reports she is an employee of Calcivis Ltd, the Company who sponsored the Clinical Study. Bruce Vernon reports he is a Calcivis Ltd employee.

## ETHICS STATEMENT

Participants gave written informed consent. The study protocol was approved by South East Scotland Research Ethics Committee 02 and was carried out in accordance with ethical principles outlined in the Declaration of Helsinki; the European Standard of BS EN ISO 14155:2011: Clinical investigation of medical devices for human subjects—good clinical practice; the International Conference on Harmonisation Good Clinical Practice Guidelines; and STROBE guidelines. The study was registered on a public database—www.clinicaltrials.gov (NCT 02780856).

## Data Availability

The data that support the findings of this study are openly available in www.clinicaltrials.gov, reference number NCT 02780856.
